# P75. Genetic engineering of T cells for increased homing to the tumor site

**DOI:** 10.1186/2051-1426-2-S2-P49

**Published:** 2014-03-12

**Authors:** M Idorn, GH Andersen, HL Larsen, JH van den Berg, Ö Met, P thor Straten

**Affiliations:** 1 Center for Cancer Immunterapi, CCIT, Herlev Universitets Hospital, Herlev Ringvej 75, 65Q9, 2730 Herlev, Denmark

## 

Adoptive cell transfer (ACT) using in vitro expanded T cells from biopsy material represents a highly promising treatment of disseminated cancer. ACT in its present form is rather crude and improvements seem within reach. Recruitment of transferred lymphocytes to the tumor site is a crucial step in ACT efficacy; however, quite few T cells actually reach the tumor site upon administration. In the present pre-clinical study we have genetically engineered T cells aiming at increasing the homing of T cells by matching expression of chemokine receptors on T cells to chemokines secreted by the tumor, thus improving anti-tumor efficacy of ACT. By PCR analysis we found that several malignant melanoma (MM) cell lines showed expression of cytokines CXCL8/IL-8, CXCL12/SDF-1 and CCL2, which was confirmed by ELISA analysis of MM conditioned medium. Taking advantage of mRNA electroporation we successfully transfected T cells with mRNA encoding the chemokine receptors CXCR2 and chimeric receptor CXCR4-R2 on the cell surface, the latter expressing the intracellular region of CXCR2 allowing expression in T cells. Work is in progress, but so far chemokine receptor CXCR2 and chimeric receptor CXCR4-R2 transfected T cells are capable of migrating towards their ligands, CXCL8 and CXCL12 respectively, in *in vitro* transwell migration assays. *In vitro* studies on the transfection and function of the CXCR4 and CCR2-R2 chimeric receptors as well as *in vivo* migration studies have been initiated, and data will be presented at the meeting.

